# Aldosterone Increases Early Atherosclerosis and Promotes Plaque Inflammation Through a Placental Growth Factor‐Dependent Mechanism

**DOI:** 10.1161/JAHA.112.000018

**Published:** 2013-02-22

**Authors:** Adam P. McGraw, Jessamyn Bagley, Wei‐Sheng Chen, Carol Galayda, Heather Nickerson, Andrea Armani, Massimiliano Caprio, Peter Carmeliet, Iris Z. Jaffe

**Affiliations:** 1 Molecular Cardiology Research Institute, Tufts Medical Center, Boston, MA (A.P.M.G., J.B., H.N., I.Z.J.); 2 Translational Immunology Science Center, Tufts Medical Center, Boston, MA (J.B.); 3 Sackler School of Graduate Biomedical Sciences, Tufts University School of Medicine, Boston, MA (W.S.C., C.G., I.Z.J.); 4 Centre for Clinical and Basic Research, Istituto di Ricovero e Cura a Carattere Scientifico San Raffaele Pisana, Rome, Italy (A.A., M.C.); 5 Laboratory of Angiogenesis and Neurovascular Link, Vesalius Research Center (VRC), Vlaams Instituut voor Biotechnologie (VIB), Leuven, Belgium (P.C.); 6 Laboratory of Angiogenesis and Neurovascular Link, VRC, Katholieke Universiteit Leuven (K.U. Leuven), Leuven, Belgium (P.C.); 7 Division of Cardiology, Tufts Medical Center, Boston, MA (I.Z.J.)

**Keywords:** atherosclerosis, growth substances, inflammation, receptors, vasculature

## Abstract

**Background:**

Aldosterone levels correlate with the incidence of myocardial infarction and mortality in cardiovascular patients. Aldosterone promotes atherosclerosis in animal models, but the mechanisms are poorly understood.

**Methods and Results:**

Aldosterone was infused to achieve pathologically relevant levels that did not increase blood pressure in the atherosclerosis‐prone apolipoprotein E–knockout mouse (ApoE−/−). Aldosterone increased atherosclerosis in the aortic root 1.8±0.1‐fold after 4 weeks and in the aortic arch 3.7±0.2‐fold after 8 weeks, without significantly affecting plaque size in the abdominal aorta or traditional cardiac risk factors. Aldosterone treatment increased lipid content of plaques (2.1±0.2‐fold) and inflammatory cell content (2.2±0.3‐fold), induced early T‐cell (2.9±0.3‐fold) and monocyte (2.3±0.3‐fold) infiltration into atherosclerosis‐prone vascular regions, and enhanced systemic inflammation with increased spleen weight (1.52±0.06‐fold) and the circulating cytokine RANTES (regulated and normal T cell secreted; 1.6±0.1‐fold). To explore the mechanism, 7 genes were examined for aldosterone regulation in the ApoE−/− aorta. Further studies focused on the proinflammatory placental growth factor (PlGF), which was released from aldosterone‐treated ApoE−/− vessels. Activation of the mineralocorticoid receptor by aldosterone in human coronary artery smooth muscle cells (SMCs) caused the release of factors that promote monocyte chemotaxis, which was inhibited by blocking monocyte PlGF receptors. Furthermore, PlGF‐deficient ApoE−/− mice were resistant to early aldosterone‐induced increases in plaque burden and inflammation.

**Conclusions:**

Aldosterone increases early atherosclerosis in regions of turbulent blood flow and promotes an inflammatory plaque phenotype that is associated with rupture in humans. The mechanism may involve SMC release of soluble factors that recruit activated leukocytes to the vessel wall via PlGF signaling. These findings identify a novel mechanism and potential treatment target for aldosterone‐induced ischemia in humans.

## Introduction

Recent clinical data has led to a new appreciation for the role of the steroid hormone aldosterone (Aldo) in the progression and adverse outcomes of atherosclerosis.^1^ Inappropriate Aldo elevation is common in rapidly growing populations at risk for cardiovascular disease and is associated with cardiovascular ischemia, yet the underlying molecular mechanisms remain to be completely understood. Although Aldo elevation is a well‐known hemodynamic consequence of congestive heart failure (CHF), inappropriate Aldo elevation is now also appreciated as a common cause of hypertension, with an incidence of 5% to 10% in patients with essential hypertension^2^ and >20% in resistant hypertension.^3^ More recently, obesity has been associated with increased Aldo,^4^ potentially because of adipocyte production of Aldo‐releasing factors.^5–6^ In these populations with high cardiovascular risk, Aldo levels are associated with adverse cardiovascular events, including myocardial infarction (MI) and stroke.^7–9^ In patients with primary hyperaldosteronism, the incidence of these events is substantially increased when compared with patients with essential hypertension and normal Aldo levels.^9^ Moreover, in patients with known atherosclerosis, higher serum Aldo levels—even within the normal range—predict a substantial increased risk of cardiovascular death.^8^

Whether Aldo is correlated with or causative for ischemia is not clear; however, clinical trials of the lipid‐modulating drug torcetrapib in patients with dyslipidemia revealed an increased risk of MI and death in patients randomly assigned to the drug, which was later associated with an off‐target increase in serum Aldo.^10–12^ Moreover, randomized clinical trials demonstrated that inhibition of Aldo production or inhibition of the receptor that mediates its effects, the mineralocorticoid receptor (MR), reduces cardiovascular ischemic events and mortality.^13–16^ Classically, Aldo activates renal MRs to promote expression of genes that enhance sodium retention, thereby increasing blood volume and blood pressure. However, the clinical benefits of MR antagonism exceed predictions based on small reductions in blood pressure, suggesting an additional blood pressure‐independent role for MRs in the complications of atherosclerosis.^17^

Atherosclerosis is a systemic vascular disease initiated by cardiovascular risk factors that cause endothelial cell (EC) damage and recruitment of leukocytes to the vascular wall,^18^ typically in regions of turbulent blood flow.^19–20^ Activated inflammatory cells within plaques release cytokines that further enhance plaque inflammation and protease activity, thereby promoting matrix degradation and plaque rupture, the cause of most MIs and strokes.^21^ Animal studies support a role for Aldo in atherosclerotic plaque formation. Aldo infusion in animal models of atherosclerosis, including the apolipoprotein E–knockout mouse (ApoE−/−), increases overall aortic plaque area with enhanced vascular and macrophage oxidative stress.^22^ Conversely, aldosterone synthase inhibitors^23^ and MR antagonists^24–25^ decrease atherosclerosis in animal models. At the cellular level, MR is expressed in vascular smooth muscle cells (SMCs), ECs, and macrophages. Activation of vascular MRs regulates gene expression^26^ and vascular function^17^; however, the role of vascular MR‐regulated genes in the pathophysiology of atherosclerosis has not been explored.

We recently identified placental growth factor (PlGF), a vascular endothelial growth factor (VEGF) family member, as an MR‐regulated vascular gene that plays a role in Aldo‐dependent vascular remodeling after injury.^27^ PlGF is a secreted factor that binds to the transmembrane VEGF type 1 receptor (VEGFR1, also known as Flt1) expressed on endothelial and inflammatory cells to promote postembryonic angiogenesis. In the setting of vascular disease, PlGF also promotes SMC proliferation^27–28^ and monocyte chemotaxis,^29^ which are fundamental components of atherosclerotic plaque development.

To identify new molecular mechanisms for the correlation between serum Aldo levels and atherosclerotic complications in patients with cardiovascular risk factors, we characterized the effects of modest Aldo increases without blood pressure elevation on the development, distribution, and composition of atherosclerotic plaques in hyperlipidemic ApoE−/− mice. We demonstrated that Aldo promotes vascular inflammatory cell recruitment and larger plaques with increased lipid content. We explored potential vascular MR‐regulated genes that might mediate this unstable plaque phenotype and identified PlGF as an Aldo‐induced factor in ApoE−/− mouse vessels. In vitro studies implicated PlGF receptor signaling in leukocyte recruitment by Aldo‐activated human coronary SMCs, and in vivo studies demonstrated a role for PlGF in Aldo‐induced vascular inflammation and atherosclerosis in this model. These studies identified new mechanisms for Aldo‐induced atherosclerosis that are potential novel targets for preventing atherosclerotic complications in humans.

## Methods

### Mouse Atherosclerosis Model

All animals were handled in accordance with National Institutes of Health standards, and all procedures were approved by the Tufts Medical Center Institutional Animal Care and Use Committee. ApoE−/− mice (C57BL/6 background, Stock #002052) and wild‐type mice (C57BL/6 background, Stock #000664) were purchased from Jackson Labs. ApoE/PlGF double‐knockout mice were generated by crossing PlGF‐knockout mice^30^ on the FVB background twice with C57BL/6 ApoE−/− mice. The resulting ApoE−/−/PlGF+/− mice on the mixed background were bred to generate ApoE−/−/PlGF−/− mice and equivalent ApoE−/−/PlGF‐intact littermates. Nine‐week‐old male mice underwent tail‐cuff blood pressure measurements (as described below), followed by implantation of a vehicle‐ (ethanol/saline) or Aldo‐infusing (6 μg/mouse per day) 4‐week osmotic minipump (Alzet model 1004). This dose of Aldo was chosen on the basis of published studies^22^ and pilot data (not shown) demonstrating resultant serum Aldo levels in the physiologically relevant range, no change in tail‐cuff blood pressure,^31^ and reproducible increases in atherosclerosis. Prior to all surgical procedures, mice were anesthetized with 1.5% isofluorane (Baxter). Animals undergoing survival surgeries received 0.05 mg/kg buprenorphine (Reckitt‐Benckiser) administered subcutaneously prior to incision and additional doses at 8‐hour intervals, if deemed beneficial. At the time of minipump implantation, mice were placed on a proatherogenic high‐fat diet (Harlan Teklad TD.88137). After 4 weeks, tail‐cuff blood pressure measurements were repeated, and mice were either euthanized for tissue collection or implanted with a second osmotic minipump for 8‐week studies.

### Serum Analyses and Blood Pressure Measurements

Over the 5 days prior to pump implantation or animal euthanization, tail‐cuff blood pressure measurements were performed using the Coda 6 System and software (Kent Scientific) by a 3‐day training and measurement protocol that we have previously described and validated.^27^ At the time of euthanization, animals were fasted for 4 hours, and blood was collected from the inferior vena cava. Animals were then perfused with phosphate‐buffered saline (PBS) at physiological pressure for 1 to 2 minutes, and tissues were collected. The aortic valve and aortic arch were embedded in optimal cutting compound (OCT) and abdominal aortas fixed in 10% neutral buffered formalin as described.^32–33^ Fasting blood glucose (Accu‐Check) was measured immediately at the time of terminal tissue harvest, and serum was subsequently assayed for insulin (ALPCO ELISA), cholesterol (RaiChem colorimetric assay), and Aldo (Siemens Health Care RIA) according to manufacturers' instructions. Circulating cytokine levels in mouse sera were measured using the Q‐plex ELISA platform (Quansys Biosciences). Serum electrolyte levels were analyzed by Idexx Radil Preclinical Services (Idexx Laboratories).

### Immunohistochemistry

Cryosections of embedded aortic roots at the site where all 3 aortic valve leaflets could be visualized and of the aortic arch where the ostia of all 3 great vessels were visible were taken at 6‐ to 10‐μm intervals. Ten‐micrometer sections were stained with Oil‐Red O (ORO), whereas sequential 6‐μm sections were stained with picrosirius red (PSR), or anti‐Mac3 antibody (BD Pharmingen) to quantify lipids, collagen, or activated inflammatory cells, respectively, along the lesser curvature of the aortic arch between the brachiocephalic and left subclavian arteries and in the aortic root at the level of the aortic valve, as described.^32–33^ Total pixels staining positive for the component of interest were normalized to overall plaque area to generate percent composition. Formalin‐fixed aortas were stripped of perivascular fat, opened longitudinally, and pinned out en face on elastomer plates. The tissues were then stained with 0.5% ORO in 85% polyethylene glycol for 3 hours, washed with PBS, and imaged. Images were collected and analyzed by a treatment‐blinded investigator using ImagePro 6.2 software (Media Cybernetics).

### Flow Cytometry

Aortic arches were stripped of adventitia and perivascular fat immediately after harvest and enzymatically digested as described.^34^ Briefly, each arch was digested in 125 U/mL collagenase type XI, 60 U/mL hyaluronidase type I‐s, 60 U/mL *DNase*1, and 450 U/mL collagenase type I (Sigma‐Aldrich) in PBS containing 20 mmol/L HEPES at 37°C for 1 hour. Cells were filtered through a 70‐μm filter to obtain a single‐cell suspension. Cells were then treated with Fc‐block (BD Pharmingen) prior to staining with APC‐Cy7‐conjugated anti‐CD45.2 (BD Pharmingen), PE‐Cy7‐conjugated anti‐CD3 (Biolegend), APC‐conjugated CD11b (Biolegend), and FITC‐conjugated CD107b (Biolegend) antibodies for 30 minutes at 4°C. Cells were analyzed on the FacsCantoII using FacsDiva software.

### Cells and Reagents

Spironolactone and Aldo (Sigma) were resuspended in DMSO for *in vitro* assays and in ethanol for *in vivo* infusion. For cell culture studies, hormones were diluted in DMEM (Gibco) and used at the indicated concentrations with corresponding vehicle controls. Immortalized human coronary artery SMCs (HCASMCs^35^) and human embryonic kidney (HEK293) cells were cultured at 37°C in DMEM supplemented with 10% bovine growth serum (BGS; HyClone). Human monocytic cells (U937) were cultured in M199 medium (Lonza) supplemented with 10% BGS. Recombinant human PlGF and VEGFR1‐blocking antibody (R&D Systems) were used at final concentrations of 25 nmol/L and 1 μg/mL, respectively. Activated U937 cells were incubated with the VEGFR 1‐blocking antibody for 10 minutes at 25°C prior to chemotaxis assays (see below).

### *Ex Vivo* Aorta Treatment, qPCR, and PlGF Measurements

ApoE−/− or WT mice were implanted with a spironolactone‐releasing drug pellet (20 mg/kg per day; Innovative Research of America) to suppress Aldo‐regulated gene expression, and 5 days later, aortas were harvested as described^26^ and incubated in DMEM containing vehicle or 100 nmol/L Aldo for 8 hours at 37°C. PlGF levels in the resulting conditioned media were measured using the mouse PlGF‐2 ELISA (R&D Systems), as described.^27^ Total aorta RNA was isolated and quantified for mRNAs encoding genes of interest by quantitative RT‐PCR (qPCR) as described.^26^

### Monocyte Chemotaxis Assays

Human coronary artery SMCs were serum‐starved in DMEM for 24 hours and then treated with the indicated hormones and vehicle controls in fresh DMEM for 18 to 24 hours. Conditioned media were then removed from the cells and centrifuged briefly at 13 000 rpm, and the supernatant was placed in the lower wells of Transwell plates (8‐μm pore size; Corning). Monocytic U937 cells were activated with 1 mmol/L 8‐bromo‐2′,3′‐cyclic adenosine monophosphate (Sigma) as described^36–37^ for 48 hours, and then 2×10^6^ cells were resuspended in DMEM and added to the upper chambers of the Transwell plates and incubated at 37°C in 5% CO_2_ for 4 hours. Inserts were fixed in methanol and stained with Giemsa stain (Sigma) per the manufacturer's instructions. Membranes were wiped free of nonadhering cells on the upper surface and mounted on glass slides. The number of migrating cells per condition was counted in 5 random fields at 10× magnification by a treatment‐blinded investigator, averaged, and normalized to the 0 nmol/L Aldo condition to generate a fold‐change in chemotactic activity.

### Statistical Analysis

Data are reported as the mean±standard error of the mean (SEM). In rare situations with extreme biological or technical variability in the histological sections, points >2 or <2 standard deviations from the mean were considered statistical outliers and were excluded from analyses (n=1 slide in the aortic arch 8‐week vehicle group in the first figure, n≤1 slide from each condition in the sixth figure). Statistical comparisons were made using SigmaPlot 11.0 (Systat Software) by *t* test or 2‐factor ANOVA where appropriate, with the Student–Newman–Keuls post test. If data were nonnormally distributed, comparisons were made using the corresponding nonparametric statistical test. *P*<0.05 was considered significant.

## Results

### Aldosterone Enhances Early Atherosclerotic Plaque Burden in Regions of Turbulent Blood Flow

**Figure 1.**
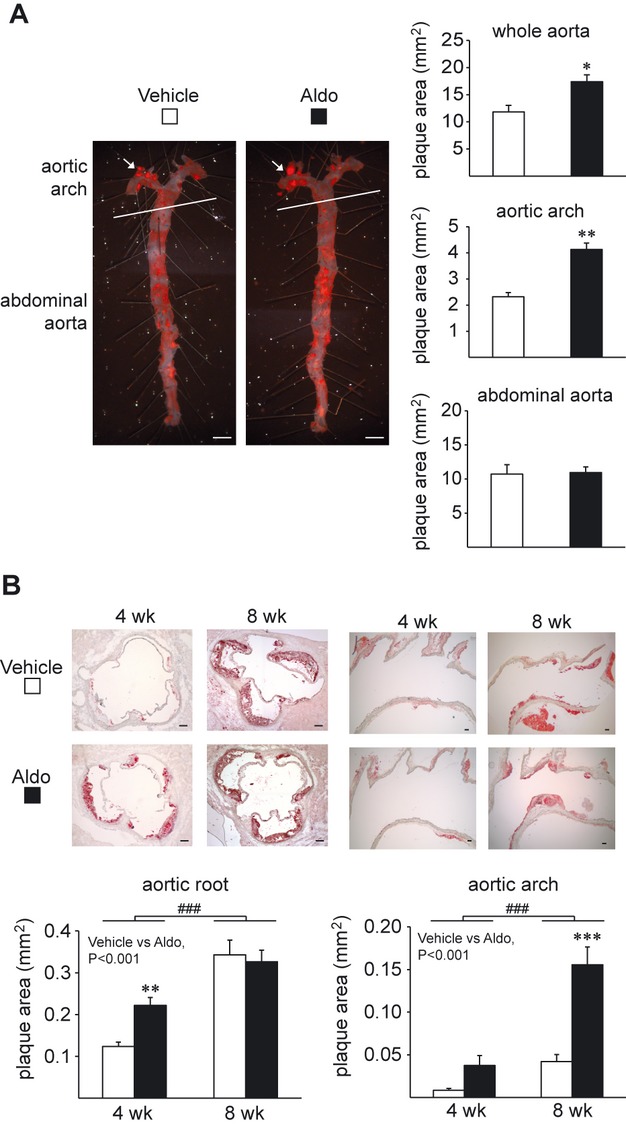
Aldo enhances early atherosclerotic plaque burden in regions with turbulent blood flow. Atherosclerotic lesions in the aortas of ApoE−/− mice after 4 or 8 weeks of a high‐fat diet and vehicle (white bars) or Aldo treatment (black bars). A, En face staining and quantification of total aorta atherosclerotic burden after 8 weeks of treatment. Scale bar=2 mm. Vehicle, n=4; Aldo, n=7. B, Histological determination of plaque area in cross‐sections of the aortic root and arch. Scale bar=0.1 mm. *P<0.05, **P<0.01, ***P<0.001 vs vehicle, ###*P*<0.001 for 4 vs 8 weeks. Aortic root: 4 weeks of vehicle, n=10; 4 weeks of Aldo, n=11; 8 weeks of vehicle, n=11; 8 weeks of Aldo, n=11. Aortic arch: 4 weeks of vehicle, n=9; 4 weeks of Aldo, n=12; 8 weeks of vehicle, n=8; 8 weeks of Aldo, n=10. Aldo indicates aldosterone; ApoE, apolipoprotein E.

ApoE−/− mice were treated with vehicle or Aldo infusion along with a high‐fat diet for 4 or 8 weeks, and the distribution of atherosclerosis in the aorta was examined. The dose was chosen to achieve a 3‐ to 5‐fold elevation in serum Aldo levels—similar to that seen in patients with cardiovascular disease—with associated electrolyte evidence of aldosterone excess (Table 1). This dose of Aldo, in the absence of high salt intake, did not significantly change traditional cardiovascular risk factors, including blood pressure, body weight, cholesterol, fasting glucose, or insulin levels, nor was there a change in heart weight, consistent with the lack of rise in blood pressure (Table 1). The short treatment durations were chosen to examine the early phases of atherogenesis to explore potential initiating mechanisms. En face staining of the whole aorta demonstrated that Aldo infusion for 8 weeks increased total aortic atherosclerosis 1.5±0.1‐fold (Figure 1A). Region‐specific integration of plaque area revealed that the Aldo effect is specific to the aortic arch, a region of turbulent blood flow, with a 1.78±0.09‐fold increase in atherosclerosis in this region and no detectable effect in the abdominal aorta, a region of laminar flow (Figure 1A).

**Table 1.** Low‐Dose Aldo Infusion Without Added Salt Does Not Change Traditional Cardiovascular Risk Factors in ApoE−/− Mice

	Vehicle (n)	Aldo (n)
Prerandomization		
Weight, g	25.1±0.4 (32)	26.0±0.4 (33)
Systolic BP, mm Hg	104.8±3 (16)	105.2±3 (19)
Diastolic BP, mm Hg	73.7±3 (16)	76.1±2 (19)
After 4 week infusion		
Weight, g	28.6±0.3 (32)	28.6±0.3 (31)
Systolic BP, mm Hg	107.1±4 (16)	103.1 ±3 (20)
Diastolic BP, mm Hg	76.6±3 (16)	72.0±3 (20)
Blood glucose, mg/dL	176±10 (10)	186±10 (10)
Serum insulin, ng/mL	0.52±0.1 (10)	0.37±0.05 (10)
Serum cholesterol, mg/dL	569±30 (16)	522±20 (16)
Heart weight, g	0.167±0.005 (15)	0.167±0.004 (15)
Serum Aldo, nmol/L	1.40±0.1 (16)	4.21±0.6** (16)
Serum sodium, mEq/L	145.9±1 (8)	149.6±0.9* (8)
Serum potassium, mEq/L	4.16±0.1 (8)	3.19±0.4* (8)
After 8 week infusion		
Weight, g	29.4±0.4 (12)	29.6±0.3 (11)
Systolic BP, mm Hg	100.5±2 (12)	105.2±3 (12)
Diastolic BP, mm Hg	67.3±2 (12)	72.0±3 (12)
Blood glucose, mg/dL	188±10 (12)	199±10 (11)
Serum insulin, ng/mL	0.46±0.1 (8)	0.32±0.1 (8)
Serum cholesterol, mg/dL	655±20 (8)	692±40 (8)
Heart weight, g	0.169±0.007 (4)	0.158±0.004 (6)
Serum Aldo, nmol/L	1.01±0.1 (8)	4.95±1* (8)

Aldo indicates aldosterone; ApoE, apolipoprotein E; BP, blood pressure.

*P<0.05, **P<0.01 Aldo vs vehicle.

Atherosclerotic burden was further evaluated in histological sections in the aortic root and the aortic arch. Aldo infusion for 4 weeks significantly increased plaque area in the aortic root 1.8±0.1‐fold (Figure 1B), with no statistically detectable change in the aortic arch or the abdominal aorta compared with vehicle (Figure 1B, data not shown). Compared with vehicle‐treated animals, Aldo infusion for 8 weeks increased plaque area in the aortic arch 3.7±0.2‐fold. As expected, plaque area in the aortic root and aortic arch was greater in the cohort of mice receiving the high‐fat diet for 8 weeks than in the group fed the high‐fat diet for 4 weeks.

### Aldosterone Promotes Unstable Plaque Phenotype With Increased Vascular and Systemic Inflammation

**Figure 2.**
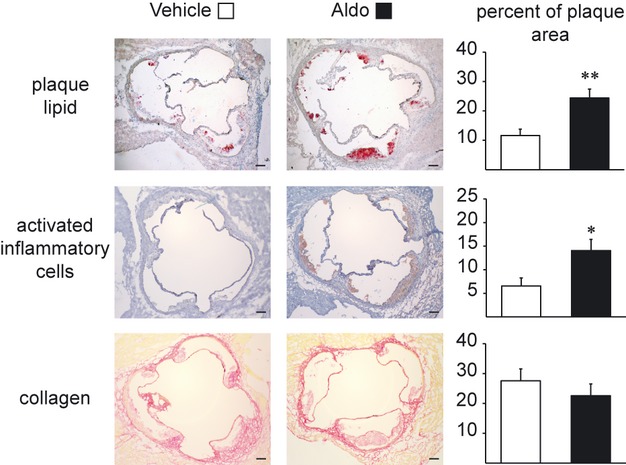
Aldo promotes an inflammatory plaque phenotype. The percentage of plaque area that stains positive for lipids (oil red O [ORO]; vehicle, n=10; Aldo, n=11), activated inflammatory cells (anti‐Mac3 antibody; vehicle, n=6; Aldo, n=7), and collagen (picrosirius red; vehicle, n=10; Aldo, n=10) was quantified in the aortic root of vehicle‐treated (white bars) or Aldo‐treated (black bars) ApoE−/− mice on a high‐fat diet for 4 weeks. *P<0.05, **P<0.01 vs vehicle. Scale bar=0.1 mm. Aldo indicates aldosterone; ApoE, apolipoprotein E.

Plaque composition was next evaluated histologically in the aortic root from ApoE−/− mice treated with vehicle or Aldo for 4 weeks, as the Aldo‐mediated increase in plaque area was most consistent in this region and with this duration of treatment. Quantification of serial sections stained with Oil Red O, anti‐Mac3 antibody, or Picrosirius Red revealed significantly increased lipid content (2.1±0.2‐fold) and activated inflammatory cell area (2.2±0.3‐fold) in plaques from Aldo‐treated mice, with no significant difference in plaque collagen content (Figure 2). This phenotype is associated with unstable plaques with an increased risk of rupture in humans.

**Figure 3.**
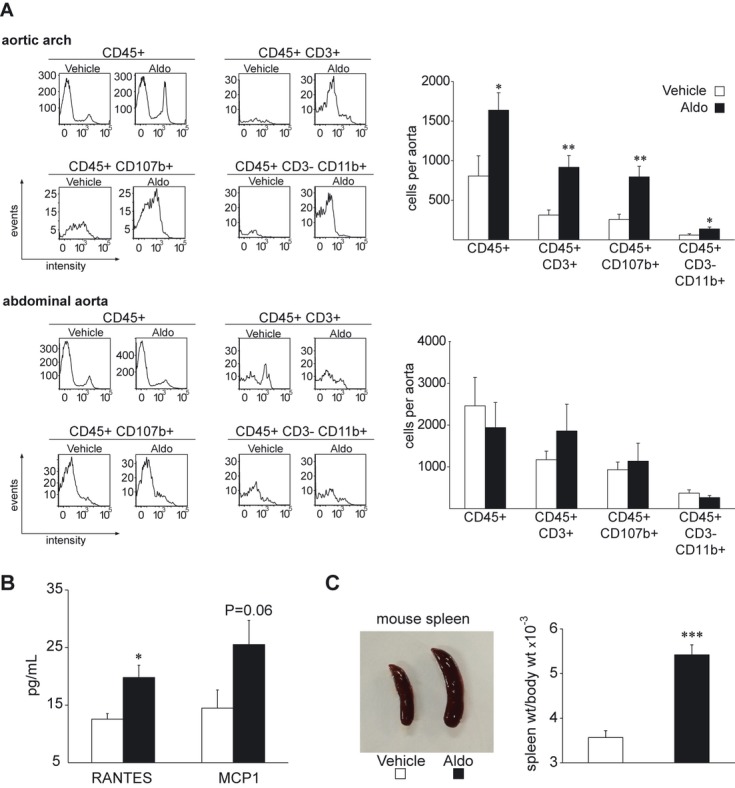
Aldo increases vascular leukocyte infiltration in atherosclerosis‐prone regions and increases systemic inflammation. ApoE−/− mice on a high‐fat diet were treated for 4 weeks with vehicle (white bars) or Aldo (black bars). A, Inflammatory cells were isolated from the aortic arch or abdominal aorta and characterized by flow cytometry. Representative histograms of the cell populations of interest are shown at left, with quantification of each shown graphically at right (n=6 per group). B, Circulating levels of RANTES and MCP1 (n=6 per group). C, Representative images of spleens (left) and quantification of spleen weight to body weight ratio (right). Vehicle, n=12; Aldo, n=14. *P<0.05, **P<0.01, ***P<0.001 vs vehicle. Aldo indicates aldosterone; ApoE, apolipoprotein E; RANTES, regulated and normal T cell secreted; MCP1, monocyte chemotactic protein‐1.

To explore the mechanism, Aldo‐induced changes in vascular inflammatory cell infiltration were measured in the aortic arch at a time that precedes significant Aldo‐induced increase in atherosclerotic burden. Total vascular cells were isolated from the aortic arches of ApoE−/− mice treated with vehicle or Aldo and a high‐fat diet for 4 weeks, and the vascular inflammatory cell infiltrate was characterized by flow cytometry. Aldo treatment increased the total number of vascular leukocytes (CD45+ cells, 2.0±0.3‐fold), T cells (CD45+CD3+ cells, 2.9±0.3‐fold), activated inflammatory cells (CD45+CD107b+ cells, 3.1±0.3‐fold), and monocytes (CD45+CD3−CD11b+ cells, 2.3±0.3‐fold) in the aortic arch (Figure 3A). There were no detectable Aldo‐dependent differences in vascular leukocyte abundance in any of these cell populations in the abdominal aortas of the same mice, an area in which Aldo did not promote subsequent plaque accumulation. Few CD45+CD3−F4/80+ cells (mature macrophages) were observed regardless of treatment or anatomical location at this early time (data not shown).

Aldo infusion for 4 weeks also resulted in signs of systemic inflammation, with increases in serum levels of the chemokine RANTES (regulated and normal T cell secreted; 1.6±0.1‐fold; Figure 3B), a nonsignificant trend toward increased MCP1 levels (monocyte chemotactic protein‐1; 1.8±0.3‐fold, *P*=0.06), and a highly significant increase in spleen size (1.52±0.06‐fold increase in spleen weight/body weight; *P*<0.001; Figure 3C). Aldo increased overall splenocyte abundance without affecting specific populations of inflammatory cells (data not shown). Serum levels of IL‐1a, IL‐1b, IL‐2, IL‐3, IL‐4, IL‐5, IL‐6, IL‐10, IL‐12, IL‐17, IFNγ, TNFα, MIP1α, and GM‐CSF were also measured and were below detectable limits (data not shown).

### Aldosterone Directly Regulates ApoE−/− Aortic Gene Expression and Increases Aortic PlGF Protein Release

**Figure 4.**
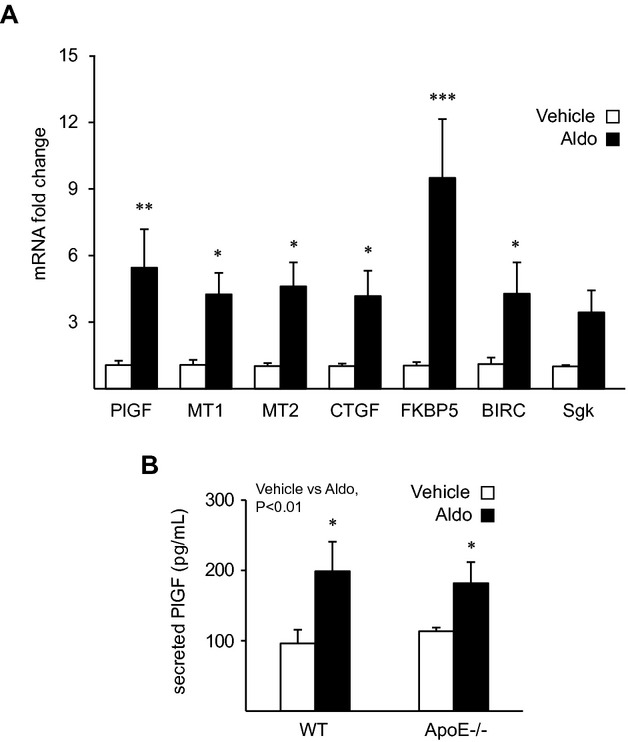
Aldo regulates vascular gene expression and placental growth factor (PlGF) secretion from ApoE−/− vessels. A, Mouse aortas were treated *ex vivo* with vehicle (n=5) or Aldo (n=5) for 8 hours, and mRNA was quantified by qRT‐PCR and expressed as the Aldo‐stimulated fold‐change for each gene. B, PlGF levels in the conditioned media from treatment of wild‐type (WT; n=3) and ApoE−/− (n=3) aortas. *P<0.05, **P<0.01, ***P<0.001 vs vehicle‐treated aortas. Aldo indicates aldosterone; ApoE, apolipoprotein E; MT1, metalothionine 1; MT2, metalothionine 2; CTGF, connective tissue growth factor; FKBP5, FK506‐binding protein 5; BIRC, baculoviral IAP repeat‐containing‐2; SGK, serum‐ and glucocorticoid‐regulated kinase; qRT‐PCR, quantitative reverse‐transcriptase polymerase chain reaction.

To explore potential mechanisms for Aldo‐induced vascular inflammation in ApoE−/− mice, we considered known Aldo‐regulated vascular genes previously identified by gene expression profiling in wild‐type mouse aortas.^26^ Messenger RNA for 7 confirmed direct vascular MR transcriptional target genes was quantified in aortas from ApoE−/− mice treated *ex vivo* with vehicle or Aldo for 8 hours (PlGF, metalothionine 1 and 2 [MT1 and MT2], connective tissue growth factor [CTGF], FK506‐binding protein 5 [FKBP5], Baculoviral IAP repeat‐containing‐2 [BIRC], and serum‐ and glucocorticoid‐regulated kinase [SGK]). Expression of RNA encoding PlGF, MT1, MT2, CTGF, FKBP5, and BIRC2 was significantly increased by Aldo in ApoE−/− vessels (Figure 4A). We and others have previously demonstrated that PlGF, MT1, MT2, and CTGF are induced by nonlaminar proatherogenic flow^38^ and that PlGF, MT1, and MT2 have enhanced Aldo regulation in the aortic arch compared with the descending aorta.^26^ Thus, we focused on PlGF because it is a secreted growth factor known to promote monocyte chemotaxis.^29^ Aldo treatment significantly enhanced PlGF protein release from the aortas of ApoE−/− mice (Figure 4B), supporting PlGF as a potential local mediator of Aldo‐induced atherosclerosis.

### Aldosterone‐Treated Human Vascular Smooth Muscle Cell Release Factors That Induce Chemotaxis Via Monocyte VEGFR1

**Figure 5.**
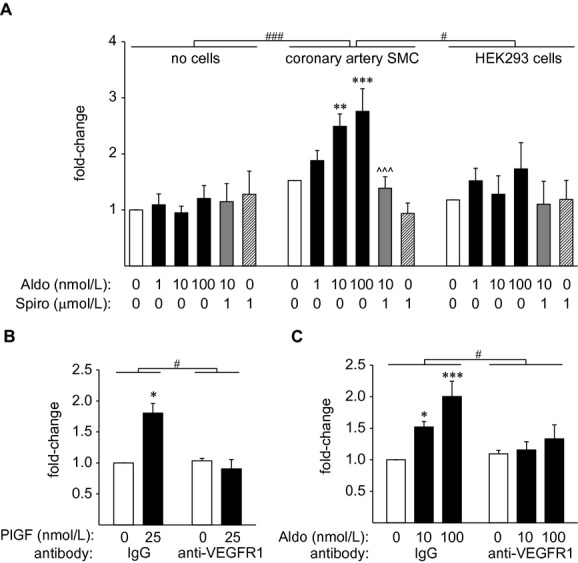
Conditioned media from Aldo‐treated human coronary SMCs promote monocyte chemotaxis through activation of monocyte VEGFR1 receptors. Monocyte chemotaxis in response to (A) conditioned media from Aldo‐ and/or spironolactone (Spiro)‐treated human coronary artery SMCs (n=6) or HEK293 cells (n=4) compared with cell‐free media (n=4). **P<0.01, ***P<0.001 vs vehicle‐treated cells, ^^^*P*<0.001 vs 10 nmol/L Aldo‐treated cells, #*P*<0.05, ###*P*<0.001 vs HCASMC cells. Monocyte chemotaxis in response to (B) PlGF (n=3) or (C) human coronary artery SMC‐conditioned media (n=5) measured in the presence of VEGFR1‐blocking or IgG control antibody. *P<0.05, ***P<0.001 vs vehicle, #*P*<0.05 vs IgG control antibody. Aldo indicates aldosterone; ApoE, apolipoprotein E; SMCs, smooth muscle cells; HEK, human embryonic kidney; VEGFR1, vascular endothelial growth factor type 1 receptor; HCASMC, human coronary artery SMC; PlGF, placental growth factor; IgG, immunoglobulin G.

Because mRNA isolated from the whole aorta proportionately comes from the more abundant SMCs in the vessel, the gene expression changes we observed in the vessel were predominantly a result of MR activation in SMCs.^26^ Therefore, we next examined, *in vitro*, the potential role of SMCs in mediating the proinflammatory effects of Aldo. Human coronary artery SMCs were treated with Aldo, and the effect of the resulting conditioned media on monocytic cell (U937 cell line) chemotaxis was investigated. In the absence of SMCs, Aldo did not influence activated monocyte migration under these conditions; however, media from Aldo‐treated SMCs induced monocyte chemotaxis in a manner that was dependent on the Aldo dose (Figure 5A). This effect of Aldo was inhibited by cotreatment of the SMCs with the MR antagonist spironolactone, supporting a new role for SMC MRs in promoting leukocyte recruitment to the vasculature. Media from similarly treated HEK293 cells did not affect U937 cell migration, and the basal chemotactic activity induced by vehicle‐treated SMCs was significantly higher than that of HEK293 cells or unconditioned media.

We next examined whether PlGF‐receptor signaling is required for Aldo‐dependent, SMC‐induced monocyte chemotaxis in vitro. As expected, PlGF alone increased monocyte migration and this effect was inhibited by preincubation of the activated monocytes with a neutralizing antibody to VEGFR1, the receptor on leukocytes through which PlGF is known to act^29^ (Figure 5B). Pretreatment of the leukocytes with the same concentration of VEGFR1‐neutralizing antibody prevented the increase in monocyte migration induced by Aldo‐treated, SMC‐conditioned media, whereas the same concentration of nonspecific control IgG antibody had no effect (Figure 5C). These data support that MR activation in SMCs recruits monocyte‐derived inflammatory cells by release of a factor that engages the leukocyte VEGFR1 receptor.

### PlGF‐Knockout Mice Are Resistant to Early Aldo‐Induced Atherosclerosis and Plaque Inflammation

**Figure 6.**
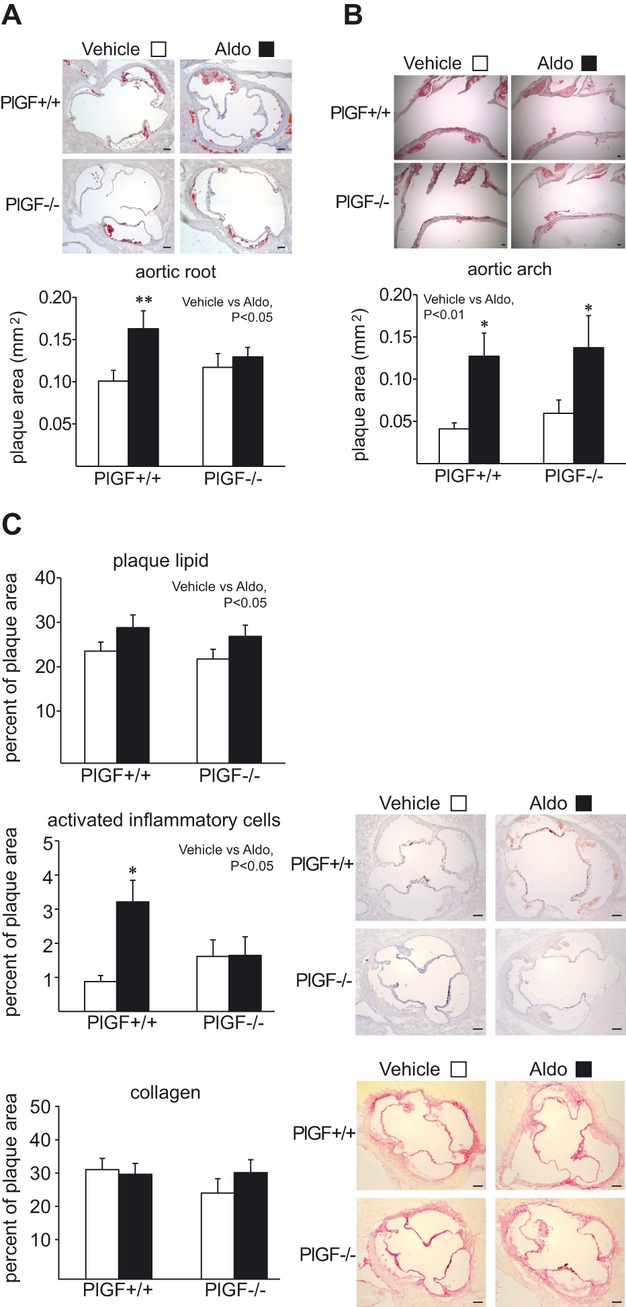
PlGF contributes to Aldo‐induced vascular inflammation and atherosclerosis *in vivo*. ApoE−/−/PlGF double knockout mice (PlGF−/−) and PlGF‐intact littermates (PlGF+/+) were administered a high‐fat diet and vehicle (white bars) or Aldo (black bars). Atherosclerotic burden in these mice was evaluated (A) in the aortic root after 4 weeks (PlGF+/+ vehicle, n=10; PlGF+/+ Aldo, n=11; PlGF−/− vehicle, n=8; PlGF−/− Aldo, n=8) and (B) in the aortic arch after 8 weeks (PlGF+/+ vehicle, n=6; PlGF+/+ Aldo, n=6; PlGF−/− vehicle, n=9; PlGF−/− Aldo, n=8). C, Quantification of aortic root plaque composition after 4 weeks of treatment. A, Plaque lipid content was quantified from ORO‐stained sections (PlGF+/+ vehicle, n=11; PlGF+/+ Aldo, n=12; PlGF−/− vehicle, n=9; PlGF−/− Aldo, n=9). Activated inflammatory cells (anti‐Mac3 antibody; PlGF+/+ vehicle, n=10; PlGF+/+ Aldo, n=12; PlGF−/− vehicle, n=9; PlGF−/− Aldo, n=8) and collagen (picrosirius red; PlGF+/+ vehicle, n=11; PlGF+/+ Aldo, n=13; PlGF−/− vehicle, n=9; PlGF−/− Aldo, n=9) were also quantified. Scale bar=0.1 mm. *P<0.05, **P<0.01 vs vehicle. PlGF indicates placental growth factor; Aldo, aldosterone; ApoE, apolipoprotein E; ORO, oil red O.

To examine in vivo the role of PlGF in Aldo‐induced atherosclerosis, we generated ApoE−/− mice that were also genetically deficient in PlGF and quantified plaque area under the same conditions that produced Aldo‐enhanced plaque burden in Figure 1B. ApoE−/−/PlGF−/− mice were treated with Aldo or vehicle and compared with ApoE−/−/PlGF‐intact littermates. Similar to purebred C57Bl6 ApoE−/− mice, Aldo infusion for 4 weeks increased aortic root plaque area 1.6±0.2‐fold in PlGF‐intact animals on this mixed C57BL6/FVB background (Figure 6A). Littermates lacking PlGF did not exhibit the Aldo‐induced increase in atherosclerosis in the aortic root under these conditions. In the aortic arch after 8 weeks of treatment, Aldo infusion increased plaque area regardless of the presence of PlGF (Figure 6B). These data support a role for PlGF in early Aldo‐induced changes in atherosclerotic plaques in the aortic root.

Next, plaque composition was evaluated in aortic root sections from ApoE−/−/PlGF double knockout mice. Aldo infusion for 4 weeks increased plaque lipid content, albeit more modestly, in these mice (Figure 6C), and this increase was not affected by PlGF genotype. Once again, no difference was observed in traditional cardiovascular risk factors between groups in this study (Table 2). Although plaques from Aldo‐treated PlGF‐intact mice exhibited a 3.7±0.3‐fold increase in activated inflammatory cell area, PlGF‐deficient animals were resistant to this Aldo‐induced plaque inflammation (Figure 6C). Neither PlGF genotype nor hormone treatment affected aortic root plaque collagen content under these conditions. PlGF deficiency did not alter the Aldo‐induced increase in spleen weight (Table 2), suggesting that the systemic proinflammatory effect of Aldo does not depend on PlGF.

**Table 2.** No Change in Traditional Cardiovascular Risk Factors With Aldo Infusion in ApoE−/−/PlGF‐Transgenic Mice

	PIGF+/+		PIGF−/−	
	Vehicle (n)	Aldo (n)	Vehicle (n)	Aldo (n)
Prerandomization				
Weight, g	28.6±0.7 (27)	28.3±0.8 (25)	27.6±0.6 (25)	28.2±0.8 (25)
Systolic BP, mm Hg	101.8±2 (9)	106.6±3 (13)	109.8±3 (9)	109.1±3 (15)
Diastolic BP, mm Hg	68.4±2 (9)	72.7±3 (13)	76.6±3 (9)	74.4±3 (15)
After 4 week infusion				
Serum Aldo*, nmol/L	1.04±0.3 (12)	3.12±0.9* (13)	0.89±0.2 (14)	4.14±2* (13)
Systolic BP, mm Hg	105.5±3 (11)	106.2±3 (13)	111.9±3 (12)	106.4±3 (12)
Diastolic BP, mm Hg	74.7±3 (11)	72.7±3 (13)	78.5±3 (12)	73.0±3 (12)
Weight, g	32.5±0.8 (27)	31.5±0.8 (31)	31.9±0.8 (25)	31.6±1 (23)
Blood glucose, mg/dL	182±10 (13)	184±10 (17)	181±10 (12)	191±10 (15)
Serum insulin, ng/mL	0.93±0.2 (12)	071±0.2 (12)	1.27±0.4 (12)	0.99±0.3 (11)
Serum cholesterol, mg/dL	806±60 (12)	842±30 (12)	821 ±50 (13)	879±50 (12)
Spleen weight/body weight (×10^3^)*	3.72±0.3 (9)	4.97±0.3* (14)	3.51 ±0.3 (6)	4.71±0.3* (8)

Aldo indicates aldosterone; ApoE, apolipoprotein E; PlGF, placental growth factor; BP, blood pressure.

* *P*<0.05: vehicle vs Aldo within genotype.

* *P*<0.05: vehicle vs Aldo main effect.

## Discussion

In summary, in a model of dyslipidemia‐induced atherosclerosis, modest and clinically relevant increases in serum Aldo resulted in (1) increased early atherosclerosis in regions of turbulent blood flow independent of changes in traditional cardiovascular risk factors including blood pressure; (2) increased plaque inflammation and lipid content, which are markers of plaque instability in humans; (3) enhanced inflammatory cell recruitment prior to increasing lesion size, specifically in atherosclerosis‐prone regions of the vasculature; and (4) increased spleen weight and circulating cytokine levels, suggesting that Aldo promotes systemic inflammation that may also contribute to atherosclerosis progression. Exploration of the mechanism revealed that (1) Aldo modulates vascular gene expression in the aortas of dyslipidemic mice and specifically increases PlGF mRNA expression and protein release; (2) MR activation in vascular SMCs induces factors that promote leukocyte chemotaxis via monocytic VEGFR1; and (3) ApoE‐knockout mice lacking the vascular Aldo‐regulated growth factor PlGF are resistant to early Aldo‐induced increases in atherosclerotic burden and plaque inflammation. These data support a new mechanism by which blood pressure–independent effects of Aldo on the vasculature promote early development of vascular inflammation and atherosclerosis.

Ample clinical data demonstrate that circulating Aldo levels are independent predictors of cardiovascular ischemia.^39–40^ Patients with primary hyperaldosteronism have a 4‐fold increased risk of stroke and a 6‐fold increased risk of myocardial infarction (MI) when compared with patients with the same degree of essential hypertension.^9^ In patients with atherosclerosis, higher serum Aldo levels—even within the normal range—predict a 2‐ to 4‐fold increase in subsequent MI or cardiovascular death,^8^ and in patients with dyslipidemia, the recent failure of the HDL‐raising drug torcetrapib was associated with an off‐target increase in serum Aldo levels that correlated with an increased rate of MI, stroke, and progression of atherosclerosis.^10–12,10–43^ Conversely, drugs that prevent Aldo production (angiotensin‐converting enzyme inhibitors or angiotensin receptor blockers) or that inhibit Aldo action (MR antagonists) reduce the risk of cardiovascular events and death out of proportion to small reductions in blood pressure (reviewed in ref. ^17^). Thus, clinical data support blood pressure‐independent effects of Aldo to promote atherosclerosis and plaque rupture in humans (reviewed in ref. ^1^).

Animal models of atherosclerosis also support a proatherogenic effect of Aldo^22,24,44^; however, the timing, distribution, and molecular mechanisms by which Aldo acts on the vasculature to contribute to atherogenesis and ischemia have not previously been explored. Here we have demonstrated that modest increases in serum Aldo in the setting of dyslipidemia increase early lesion formation and plaque lipid and inflammatory cell content. In humans, this same phenotype is vulnerable to plaque rupture, the cause of most heart attacks and strokes.^21^ Prior studies have shown that deletion of 11‐β‐hydroxysteroid dehydrogenase type 2 (11βHSD2) in ApoE−/− mice, which results in chronic cortisol‐mediated activation of MRs, promotes formation of larger plaques with a similar unstable phenotype.^45^ Because of significant hypertension in the 11βHSD2‐knockout mouse, the direct, blood pressure–independent effects of Aldo in this model are difficult to interpret. We specifically infused Aldo to achieve serum levels that did not raise blood pressure using a well‐validated tail‐cuff blood pressure method similar to that used in prior studies in ApoE−/− mice on a high‐fat diet.^31^ A modest rise in blood pressure was demonstrated by Keidar et al^22^ in ApoE−/− mice infused with Aldo at similar rates; however, that study was performed on a normal‐fat diet with unclear sodium content, resulting in higher serum Aldo levels, and used a tail‐cuff blood pressure method that lacked a training period. Thus, our study demonstrates, for the first time, that Aldo promotes early plaque development with an unstable phenotype independent of changes in blood pressure, supporting direct vascular effects of Aldo in the setting of atherosclerosis. Consequently, Aldo‐mediated increases in vulnerable plaques may explain both the reduction in cardiovascular ischemic events in patients receiving MR antagonists and the increased incidence of myocardial infarction and death in patients with higher aldosterone levels or in the treatment arm of the torcetrapib trials.

Aldo infusion for 4 to 8 weeks in ApoE−/− mice increased atherosclerotic burden in the aortic root and aortic arch without a detectable effect on plaque area in the abdominal aorta. One potential explanation for this finding is that the proatherosclerotic effects of Aldo are localized specifically to regions of turbulent blood flow. Regions such as the aortic root and aortic arch are susceptible to low wall shear stress, which triggers endothelial dysfunction, vascular oxidative stress, and leukocyte adhesion, which are important initiating steps in plaque formation.^19–20^ A recent study demonstrated that surgically‐induced RAAS activation in mice combined with low vascular shear stress produces plaque rupture and supports an interaction between RAAS activation and shear stress.^46^ In addition, the aortic arch exhibits greater Aldo‐dependent regulation of multiple proatherogenic genes^38^, including *PlGF*, at least partly because of increased oxidative stress.^26^ These findings support a new molecular mechanism for region‐specific variations in Aldo‐mediated atherosclerosis.

Although Aldo treatment for 4 weeks increased aortic root plaque area, after 8 weeks of treatment plaque size was unchanged between treatment groups, suggesting that Aldo contributes specifically to early atherogenesis. Aldo treatment increases the number of vascular monocytes and T cells in the aortic arch before lesions appear, with no change in these inflammatory cell populations in the descending aorta. Thus, vascular inflammatory cell recruitment and activation in response to Aldo occur prior to plaque development, specifically in regions that are predisposed to Aldo‐induced atherosclerosis, supporting the potential for vascular leukocyte recruitment to play a role in the mechanism of early Aldo‐induced atherosclerosis. In humans, plaque rupture tends to occur in smaller, nonobstructive lesions. By enhancing the development of early and more inflamed plaques, Aldo might promote the adverse consequences of atherosclerosis without affecting the ultimate size or phenotype of more mature and stable plaques.

Although MR activation in ECs likely contributes to atherosclerosis by upregulating expression of intracellular adhesion molecule 1 (ICAM1), thereby enhancing leukocyte‐EC adhesion,^47^ much less is known about the role of SMCs during early atherosclerosis development. In advanced lesions, SMCs stabilize plaques by synthesizing collagen and other matrix proteins.^48^ Prior to lesion formation, however, vascular SMCs have been shown to upregulate expression of adhesion molecules and cytokines,^49–50^ particularly in the setting of proatherogenic hemodynamic flow.^50^ Thus, SMCs could contribute to leukocyte recruitment during early atherogenesis, while adopting more protective mechanisms as the disease progresses. Here we have demonstrated that conditioned media from human coronary artery SMCs promote monocyte chemotaxis (even without Aldo), supporting a novel role for SMCs in recruiting leukocytes to specific locations in the vasculature. Moreover, when SMCs are exposed to Aldo, there is increased production of soluble factors that promote monocyte chemotaxis in vitro. This effect is blocked when SMCs are cotreated with the MR antagonist spironolactone, supporting the idea that SMC MRs directly contribute to atherosclerosis. Future studies with mice specifically deficient in SMC MRs will be needed to formally test this possibility in vivo.

We focused on PlGF because this factor was previously shown to be regulated by Aldo in the aorta independent of the endothelium,^27^ to be upregulated by Aldo in the aortic arch to a greater extent than in the abdominal aorta,^26^ to be produced by SMCs in response to RAAS activation,^51^ to recruit inflammatory cells,^52^ and to contribute to plaque inflammation and early lesion development in the setting of atherosclerosis.^53–55^ Mechanistically, PlGF is transcriptionally regulated in the vasculature by Aldo likely via an Aldo‐sensitive DNA element in the PlGF upstream DNA sequence.^27^ PlGF also induces monocyte chemotaxis by activation of leukocyte VEGFR1 receptors,^29^ and macrophages isolated from mice carrying a mutation that inactivates VEGFR1 signaling exhibit reduced chemotactic activity in response to PlGF.^56–57^ Here we have shown that blockade of monocyte VEGFR1 receptors prevents Aldo‐dependent, SMC‐induced chemotaxis and that ApoE/PlGF double‐knockout mice are resistant to the Aldo‐induced increase in early aortic root plaque burden and inflammation. These studies implicate MR‐mediated upregulation of PlGF in the ApoE−/− aorta as an important mechanism of Aldo‐dependent atherogenesis and vascular inflammation. Additional data support the potential for this mechanism to be relevant in humans. In aortic tissue explanted from patients with atherosclerosis, Aldo enhanced—and MR antagonism inhibited—PlGF and VEGFR1 expression.^27^ In patients undergoing carotid endarterectomy, PlGF expression correlated with the degree of carotid artery macrophage infiltration, expression of inflammatory markers, and symptoms of clinical plaque instability.^58^ In addition, pharmacologic manipulations that decreased serum PlGF in patients presenting with acute chest pain correlated with improved outcomes.^59^ These data support the new model that SMC MRs induce PlGF expression locally in areas of turbulent blood flow, resulting in enhanced vascular leukocyte recruitment and thereby contributing to Aldo‐associated complications of atherosclerosis in humans. This mechanism could represent a novel clinical target to prevent the adverse consequences of atherosclerosis in high‐risk populations.

There are several limitations to this study that should be considered. These studies were all performed in male mice. Because cardiovascular risk differs by sex in humans, the role of Aldo in atherogenesis and vascular inflammation in female mice deserves further examination. Previous studies showed that female ApoE−/−/PlGF−/− mice exhibit reduced plaque size compared with ApoE−/−/PlGF‐intact littermates after 10 weeks of a high‐fat diet.^54^ Here we observed no difference in atherosclerosis burden in vehicle‐treated male mice lacking PlGF after 4 weeks of high‐fat feeding. Thus, the contribution of PlGF to plaque development independent of Aldo may vary depending on sex, duration of hyperlipidemia, and other factors that remain to be explored. Here we focused on the contribution of vascular SMC MRs and of PlGF to the mechanism of Aldo‐enhanced atherosclerosis. Although ApoE/PlGF double‐knockout mice are resistant to early Aldo‐induced vascular inflammation, they are not resistant to Aldo‐enhanced plaque lipid content or increased aortic arch plaque area after 8 weeks, indicating that other mechanisms contribute to these effects of Aldo. Indeed, we demonstrated that Aldo regulates multiple proatherogenic vascular genes in addition to *PlGF*,^26^ and these and other vascular MR target genes likely also contribute to these processes and deserve further study. MRs in cell types other that SMCs also contribute to atherosclerosis. MR activation in ECs enhances leukocyte–EC adhesion,^47^ whereas inappropriate activation of perivascular adipocyte MRs promotes adipocyte dysfunction, inflammation, and oxidative stress.^60^ Macrophages and T cells also express MRs.^61^ Although we did not observe direct effects of Aldo on macrophage chemotaxis in vitro, mice lacking macrophage MRs have altered macrophage activation and decreased cardiovascular remodeling in response to mineralocorticoid/salt‐induced hypertension without altered macrophage infiltration into tissues.^62–63^ In the ApoE−/− model, we demonstrated that Aldo increases spleen size and serum levels of the chemokine RANTES, supporting effects of Aldo on the systemic immune response that could also contribute to atherosclerotic progression. Although it is known that ApoE−/− mice have enlarged spleens,^64^ a role for Aldo in splenomegaly in these mice has not been previously described. The effects of Aldo on spleen size persist in PlGF‐KO mice, consistent with a distinct mechanism for Aldo effects on the immune response. Thus, Aldo‐enhanced atherogenesis is likely a cumulative and complex process mediated by multiple interacting cell types. The role of MRs in these various extrarenal cells in promoting cardiovascular disease warrants further exploration.

In summary, we have demonstrated that Aldo promotes early atherogenesis and enhances region‐specific vascular inflammatory cell recruitment, resulting in an unstable plaque phenotype that may lead to cardiovascular ischemia upon rupture. SMC MR‐dependent PlGF signaling is a potential new mechanism for Aldo‐induced inflammatory cell recruitment contributing to larger, more inflamed plaques in vivo. This study supports a new mechanism for the association of serum Aldo levels with acute cardiovascular ischemia in humans and for the vascular protective effects of renin–angiotensin–aldosterone system antagonists and identifies new potential targets to prevent cardiovascular ischemia.
